# Identification of a novel circ_0018289/miR-183-5p/TMED5 regulatory network in cervical cancer development

**DOI:** 10.1186/s12957-021-02350-y

**Published:** 2021-08-17

**Authors:** Heng Zou, Huijia Chen, Shuaibin Liu, Xiaoling Gan

**Affiliations:** 1 The Center for Reproductive Medicine, Obstetrics and Gynecology Department, The Second Affiliated Hospital of Chongqing Medical University, Chongqing, 400010 China; 2 Department of Obstetrics and Gynecology, The Second Affiliated Hospital of Chongqing Medical University, No.74 Linjiang Road, Yuzhong District, Chongqing, 400010 China

**Keywords:** Cervical cancer, circ_0018289, miR-183-5p, TMED5, Angiogenesis

## Abstract

**Background:**

Circular RNAs (circRNAs) are increasingly implicated in regulating human carcinogenesis. Previous work showed the oncogenic activity of circ_0018289 in cervical cancer. However, the molecular basis underlying the modulation of circ_0018289 in cervical carcinogenesis is still not fully understood.

**Methods:**

The levels of circ_0018289, microRNA (miR)-183-5p, and transmembrane p24 trafficking protein 5 (TMED5) were measured by quantitative real-time polymerase chain reaction (qRT-PCR) or western blot assay. Ribonuclease (RNase) R and subcellular localization assays were used to characterize circ_0018289. Cell proliferation was detected by the Cell Counting Kit-8 (CCK-8) and 5-ethynyl-2′-deoxyuridine (Edu) assays. Cell apoptosis and tube formation were assessed by flow cytometry and tube formation assays, respectively. A dual-luciferase reporter assay was performed to confirm the direct relationship between miR-183-5p and circ_0018289 or TMED5. The role of circ_0018289 in tumor growth was gauged by mouse xenograft experiments.

**Results:**

Circ_0018289 was overexpressed in cervical cancer tissues and cells. Circ_0018289 silencing impeded cell proliferation, enhanced cell apoptosis, and suppressed angiogenesis in vitro, as well as diminished tumor growth in vivo. Mechanistically, circ_0018289 targeted and regulated miR-183-5p by binding to miR-183-5p, and circ_0018289 regulated cervical cancer development and angiogenesis partially through miR-183-5p. Moreover, TMED5 was directly targeted and inhibited by miR-183-5p through the perfect complementary sites in TMED5 3′UTR, and TMED5 knockdown phenocopied miR-183-5p overexpression in suppressing cervical cancer development and angiogenesis. Furthermore, circ_0018289 induced TMED5 expression by competitively binding to shared miR-183-5p.

**Conclusion:**

Our observations identified the circ_0018289/miR-183-5p/TMED5 regulatory network as a novel molecular basis underlying the modulation of cervical carcinogenesis.

**Supplementary Information:**

The online version contains supplementary material available at 10.1186/s12957-021-02350-y.

## Introduction

Among women, cervical cancer ranks fourth for cancer mortality throughout the world [1]. Early-stage cervical cancer is curable, and more advanced tumors cannot be successfully treated despite the use of therapeutic methods that combine surgery and radiochemotherapy [2, 3]. Because the molecular determinants of cervical cancer are still not thoroughly understood, developing more effective therapies remains difficult. Hence, there is an urgent need to know the molecular basis of cervical carcinogenesis.

Covalently closed circular RNAs (circRNAs) are naturally occurring RNA biomolecules without 3′ polyadenylate tails or 5′ caps [4]. Insights into the potential functions of circRNAs on gene expression by inhibiting microRNA (miRNA) activity highlight their essential involvement in the biological basis of cancers, including cervical cancer [5–7]. For instance, Song et al. illuminated that circ_101996, an overexpressed circRNA in cervical cancer, worked as a strong promoter of the disease via miR-8075-dependent regulation of TPX2 microtubule nucleation factor [8]. Tang et al. uncovered that circ_0000515 contributed to cervical carcinogenesis by binding to miR-326 to induce ETS transcription factor ELK1 [9]. As for circ_0018289, generated from the exons of synaptotagmin 15 (SYT15) mRNA, it was demonstrated to be upregulated in cervical cancer, highlighting its potential as a promising biomarker for the outcome of patients [10]. Previous work also showed the oncogenic activity of circ_0018289 in cervical cancer depending on the modulation of miR-497 [11]. Nevertheless, our understanding of the molecular basis underlying the modulation of circ_0018289 in cervical carcinogenesis has remained incomplete.

Here, our data supported the tumor-promoting property of circ_0018289 in cervical cancer. We identified that circ_0018289 bound to miR-183-5p, a potent suppressor in cervical cancer [12, 13]. Furthermore, we demonstrated that the circ_0018289/miR-183-5p axis-mediated transmembrane p24 trafficking protein 5 (TMED5) expression impacted cervical carcinogenesis and angiogenesis.

## Materials and methods

### Bioinformatics

The putative miRNAs that directly bound to circ_0018289 were searched by the computer algorithm circInteractome (https://circinteractome.nia.nih.gov/). MiRNA target prediction of human 3′untranslated region (3′UTR) transcripts was performed using the computational prediction software starBase (http://starbase.sysu.edu.cn/). Association between TMED5 level and the prognosis of cervical cancer patients was downloaded from the GEPIA database (http://gepia.cancer-pku.cn/).

### Human samples and cells

Fresh-frozen samples of cervical cancer (*n* = 50) and matched normal cervical tissues (*n* = 50) were obtained with written informed consent from individuals undergoing cervical resection in the Second Affiliated Hospital of Chongqing Medical University. Samples were analyzed for the expression levels of circ_0018289, miR-183-5p, and TMED5. Approval to collect human samples was granted by the Ethical Committee of the Second Affiliated Hospital of Chongqing Medical University.

Human ectocervical Ect1/E6E7 cells, SiHa and HeLa cervical cancer cells, and human umbilical vein endothelial cells (HUVECs) were originally from American Type Culture Collection (ATCC, Manassas, VA, USA) and cultivated at 37 °C in 5% CO_2_ under standard conditions provided by ATCC. SiHa and HeLa cells were grown in EMEM with 10% FBS and 1% penicillin–streptomycin (all from Gibco, Tokyo, Japan). Ect1/E6E7 cells were cultured in serum-free keratinocyte medium with 0.05 mg/mL bovine pituitary extract, 0.1 ng/mL human recombinant EGF, 1% penicillin–streptomycin, and 0.4 mM calcium chloride (all from Gibco). HUVECs were maintained in F-12 K medium containing 10% FBS, 0.1 mg/mL Heparin, 0.05 mg/mL ECGs, and 1% penicillin–streptomycin (all from Gibco).

### Plasmids

Human TMED5 (Accession: NM_001167830.2) coding sequence (lacking the 3’UTR region) incorporated with BamH I and EcoR V sites in two terminals and the scrambled control sequence were synthesized by BGI (Shenzhen, China) and individually inserted into the pcDNA3.1 vector (Invitrogen, Wesel, Germany) opened with BamH I and EcoR V sites to generate TMED5 overexpression plasmid (pcDNA-TMED5) and negative control (pcDNA). The fragments of human circ_0018289 and TMED5 3′UTR encompassing the target sequence for miR-183-5p or mutated target sites were synthesized by BGI and individually cloned into the 3′UTR of *Renilla* luciferase in a psiCHECK-2 vector (Promega, Milano, Italy) opened with Xho I and Pme I sites to generate luciferase reporter constructs.

### Transient transfection of cells

SiHa and HeLa cells were plated at 5 × 10^5^ cells/well in 24-well cell culture dishes 18 h before transfection with synthetic circ_0018289-selected siRNA (si-circ_0018289) or control shRNA (si-NC) at 100 nM, mature miR-183-5p mimic or mimic control (miR-NC mimic) at 50 nM, or the antisense oligonucleotide of the mature miR-183-5p sequence designed to silence miRNA (anti-miR-183-5p) or nontarget control sequence (anti-miR-NC) at 50 nM using the HiPerFect transfection reagent as per the manufacturing recommendations (Qiagen, Tokyo, Japan). The details of all oligonucleotides (Ribobio, Guangzhou, China) were in Supplement Table 1. For TMED5 overexpression, 5 × 10^5^ SiHa and HeLa cells seeded in each well of 24-well cell culture dishes were transiently transfected with pcDNA-TMED5 or pcDNA plasmid at a final dose of 200 ng using Lipofectamine 3000 as recommended by the manufacturers (Invitrogen). Transfected cells were collected for further assays 2 days post-transfection.

### Quantitative real-time polymerase chain reaction (qRT-PCR)

Total RNA, inclusive of the small RNA fraction, was prepared from homogenized tissues and cultured cells with Trizol reagent as recommended by the manufacturers (Invitrogen). For circ_0018289 and mRNA quantification, cDNA was randomly primed with ReverTra Ace RT Kit (Toyobo, Tokyo, Japan) in a 25-μL reaction containing 500 ng of extracted RNA. qRT-PCR with iQ SYBR Green (Bio-Rad, Munich, Germany) and amplification primers (shown in Supplementary Table 1) was done on a PCR machine (Rotorgene 6000, Qiagen) with the following conditions: after a denaturation time of 10 min at 95 °C, 40 cycles at 95 °C for 20 s and at 60 °C for 1 min. For miR-183-5p quantification, cDNA preparation and qRT-PCR were carried out as above, using miScript RT Kit and SYBR Green Kit (all from Qiagen), respectively. Fold changes in gene and miRNA expression were determined by the 2^−ΔΔCt^ method, normalizing the results to normal controls and expression of β-actin or U6. ΔCt was calculated by subtracting the Ct values of β-actin or U6 from the Ct values of the gene of interest. ΔΔCt was then calculated by subtracting ΔCt value of the control from ΔCt of the sample.

### Ribonuclease (RNase) R assay

This experiment was performed by incubating 3 μg of extracted RNA with 10 U of RNase R (Epicenter Biotechnologies, Madison, WI, USA) at 37 °C for 20 min. After being purified by the Trizol RNA Purification Kit (Invitrogen), RNA was analyzed by qRT-PCR analysis for quantification of circ_0018289 and SYT15 mRNA levels as above.

### Subcellular localization assay

For the preparation of nuclear and cytoplasmic RNA of SiHa and HeLa cells, a Cytoplasmic and Nuclear RNA Purification Kit was used as described by the manufacturers (Norgen Biotek, Thorold, ON, Canada). For localization analysis, U6 and glyceraldehyde-3-phosphate dehydrogenase (GAPDH) served as the nuclear and cytoplasmic control, respectively. The isolated RNA was analyzed by qRT-PCR analysis for assessment of circ_0018289, U6, and GAPDH levels.

### Cell proliferation assay

For the evaluation of cell proliferation, 5-ethynyl-2′-deoxyuridine (Edu) labeling and Cell Counting Kit-8 (CCK-8) assays were performed. Briefly, for Edu assay, transfected cells were incubated with Edu solution (50 μM, Ribobio) for 2 h, and subsequently, the Edu-labeled cells were stained with 1 × Apollo567 (Ribobio) for 30 min. After being staining with 4′,6-diamidino-2-phenylindole (DAPI, Invitrogen) for nuclei staining, the cells were observed under a BZ-8000 fluorescence microscope (Keyence, Osaka, Japan). The ratio of Edu-positive nuclei (red) to total nuclei (blue) was determined as the proliferation rate of transfected cells in 10 randomly captured fields per well. For CCK-8 assay, ~ 2500 transfected cells were plated into each well of 96-well cell culture dishes and incubated in complete medium at 37 °C. The cells were evaluated for potential growth every 24 h using the CCK-8 solution (10 μL per well) as per the manufacturing guidance (Dojindo, Kumamoto, Japan). After addition of CCK-8 solution, a 2-h incubation was allowed at 37 °C. A 96-well spectrometer (BMG Labtech, Ortenberg, Germany) was used to measure the absorption with a 450 nm filter.

### Flow cytometry for cell apoptosis

Following the wash in cold phosphate-buffered saline (Solarbio, Beijing, China), transfected cells (5 × 10^5^) were incubated with propidium iodide (PI, 50 mg/mL, Invitrogen) and Annexin V-fluorescein isothiocyanate (10 μL, Annexin V-FITC, BD Pharmingen, Heidelberg, Germany) as per the accompanying protocols. Following a 15-min incubation in the dark, data were analyzed within 1 h using an Epics XL-MCL flow cytometer (Beckman Coulter, Fullerton, CA, USA) with FlowJo 9.1 software (Tree Star, Ashland, OR, USA). Apoptotic cells were defined as the population that was positive for Annexin.

### Tube formation assay

Transfected cells (1 × 10^5^ cells per well) were seeded in 24-well cell culture dishes and incubated in complete medium at 37 °C. When the cells reached approximately 70% confluence, the complete medium was replaced by the serum-free EMEM medium. Following a 48-h culture at 37 °C, the conditional medium was harvested by centrifugalization. Subsequently, about 2 × 10^4^ HUVECs were suspended in 200 μL of the conditional medium and then plated in 24-well cell culture dishes precoated with Matrigel Basement Membrane Matrix (BD Biosciences, Cowley, UK). Images of tube morphology were photographed using an inverted 100 × microscope (Keyence), and the tube formation was monitored every 6 h. The formed tubes were determined by counting the number of meshes using ImageJ software.

### Western blot

Total protein was prepared from homogenized tissues and cultured cells using RIPA buffer (Solarbio) plus protease inhibitors (Roche, Mannheim, Germany) and quantified by the BCA method (Thermo Fisher Scientific, Monza, Italy). Immunoblots were carried out with the isolated protein as described [14]. Equal amounts of protein were resolved by electrophoresis on Criterion™ TGX™ precast 10% gels (Bio-Rad), transferred to nitrocellulose membranes (BD Biosciences), and probed with antibodies against B cell lymphoma-2 (Bcl-2, ab182858, dilution 1:2000), vascular endothelial growth factor A (VEGFA, ab1316, dilution 1:1000), fibroblast growth factor 2 (FGF2, ab92337, dilution 1:3000), Bcl-2 associated X (Bax, ab182733, dilution 1:2000), TMED5 (ab254795, dilution 1:1000), and β-actin (ab8227, dilution 1:3000) from Abcam (Cambridge, UK). The horseradish peroxidase-conjugated IgG (ab205718, dilution 1:5000, Abcam) was used as the secondary antibody. Chemiluminescence was achieved by the incubation of Clarity ECL substrate (Bio-Rad). Protein blots were scanned and quantified using AIDA software (Raytek, Sheffield, UK).

### Dual-luciferase reporter assay

Luciferase reporter constructs (200 ng) were transfected using Lipofectamine 3000 into SiHa and HeLa cells seed in 24-well cell culture dishes (5 × 10^5^ cells/well) together with synthetic miR-183-5p mimic or control mimic at 50 nM. Luciferase activity was gauged after 2 days using a 10 μL of cell lysates with the Luc-Pair miR Luciferase Assay Kit (GeneCopoeia, Rockville, MD, USA) on a luminometer (Promega) as per the accompanying guidance.

### Generation of stable circ_0018289 silencing cell line

Lentiviruses expressing shRNA-circ_0018289 (sh-circ_0018289) or scrambled shRNA (sh-NC) were provided by Geneseed (Guangzhou, China) and used to infect SiHa cells in media containing polybrene (8 μg/mL, Solarbio). A stable cell line was selected using 2 mg/mL of puromycin for over 7 days.

### Mouse xenograft experiments

All experiments with mice conformed to the protocols approved by the Animal Use Committee of the Second Affiliated Hospital of Chongqing Medical University, and all mice were cared following National Institutes of Health guidelines. For the formation of xenograft tumors, 4- to 5-week-old female BALB/c nude mice (Gempharmatech Biotechnology Co., Ltd., Jiangsu, China) were implanted subcutaneously with sh-NC- or sh-circ_0018289-transduced SiHa cells (5 × 10^6^ cells per mouse) in 200 μL of cell culture medium (*n* = 6 mice per group). Tumor growth was monitored weekly by caliper measurements and tumor volume was determined by the equation *D* × *d*^2^/2, where *D* was the longest diameter of the tumor and *d* was the shortest diameter. Mice were sacrificed at day 35 after cell implantation with CO_2_ overdose and the tumors were harvested for weight and expression analysis. Proliferation of tumors was evaluated with paraffin embedded tumor sections (4 μm) by immunohistochemistry using a monoclonal antibody against Ki67 (ab16667, Abcam, dilution 1:200), Vectastain ABC Kit (Vector Laboratories, Burlingame, CA, USA), and 3,3′-diaminobenzidine (DAB) substrate (Vector Laboratories) as described [15].

### Statistical analysis

For descriptive statistics, data were presented as mean ± standard derivation from at least 3 independent biological replicates (performed in quadruplicate). Statistical analysis was done on the Prism 7 software (GraphPad, San Diego, CA, USA). *P* values were determined by Student’s two-tailed *t*-test (comparison in two groups), Mann–Whitney *U* test (comparison in human samples), or one-way analysis of variance with Tukey–Kramer multiple comparison test (comparison in multiple groups), with *P* < 0.05 accepting significance. The Pearson’s correlation coefficients were used to determine the correlations among circ_0018289, miR-183-5p, and TMED5 expression levels in human tumors.

## Results

### Circ_0018289 was overexpressed in cervical cancer

**Fig. 1:**
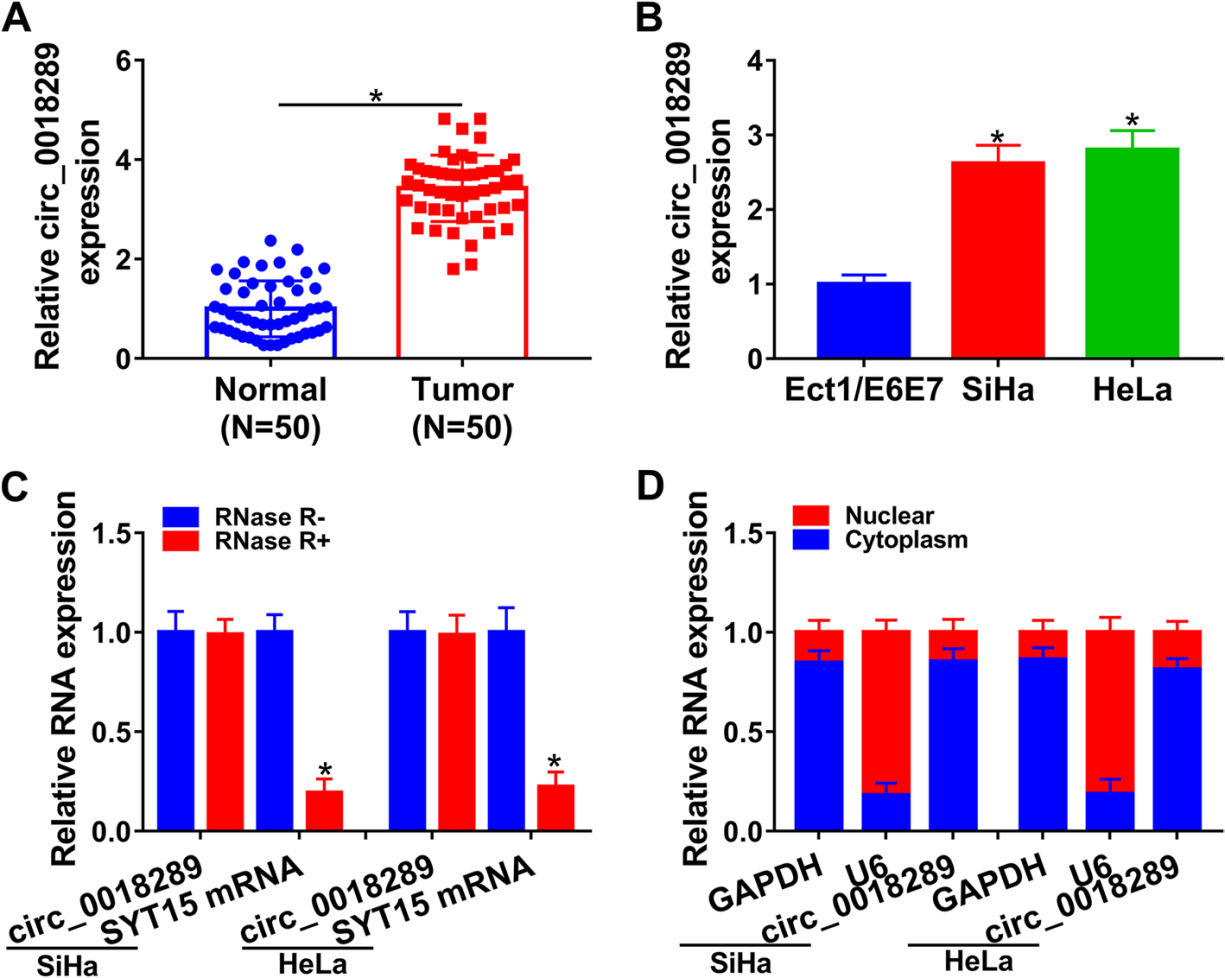
Circ_0018289 expression was increased in cervical cancer. **A** qRT-PCR analysis of circ_0018289 in 50 primary tumor samples and 50 paired normal tissues from the same patients. **B** Circ_0018289 expression by qRT-PCR analysis in Ect1/E6E7, SiHa, and HeLa cells. **C** RNase R assay in SiHa and HeLa cells. **D** Subcellular localization assay in SiHa and HeLa cells. *P < 0.05

To evaluate the role of circ_0018289 in cervical cancer, we firstly analyzed its expression in 50 primary tumor samples by qRT-PCR analysis. Circ_0018289 was markedly upregulated in tumor samples compared with paired normal tissues (Fig. 1A). In agreement with tumor samples, cancer cells showed higher levels of circ_0018289 compared to ectocervical Ect1/E6E7 cells (Fig. 1B). We then determined the stability of circ_0018289 in SiHa and HeLa cells by RNase R assay. Circ_0018289, rather than the corresponding linear SYT15 mRNA, was resistant to RNase R (Fig. 1C). Furthermore, circ_0018289 was mainly present in the cytoplasm of SiHa and HeLa cells, which was confirmed by subcellular localization assay (Fig. 1D).

### Circ_0018289 silencing impeded cell proliferation and enhanced cell apoptosis in vitro

**Fig. 2:**
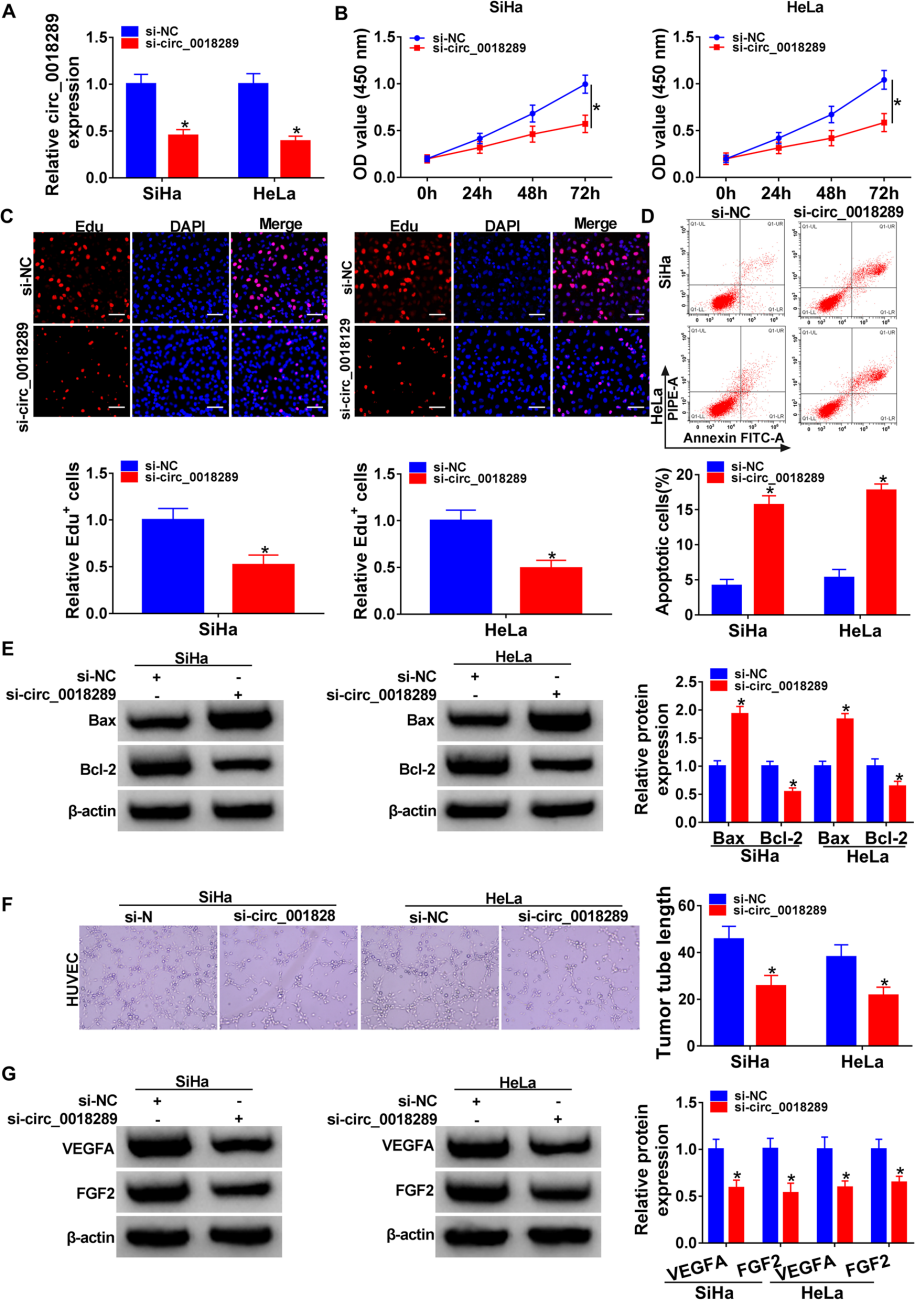
Circ_0018289 silencing suppressed cervical carcinogenesis and angiogenesis in vitro. SiHa and HeLa cells were transfected with si-circ_0018289 or si-NC. **A** qRT-PCR analysis of circ_0018289 in transfected cells. **B** CCK-8 assay showing cell proliferation ability. **C** Representative images depicting a cell proliferation assay and cell proliferation by Edu assay. Scale bars, 100 μm. **D** Representative images depicting a cell apoptosis assay and cell apoptosis by flow cytometry. **E** Western blot showing the levels of Bax and Bcl-2 in transfected cells. **F** Representative images depicting a tube formation assay performed with human HUVECs pre-treated with the conditional medium of transfected cells. 100 × magnification. **G** Western blot showing the expression levels of VEGFA and FGF2 in transfected cells. *P < 0.05

To elucidate the functional action of circ_0018289 in cervical carcinogenesis, we used siRNA-circ_0018289 (si-circ_0018289) in SiHa and HeLa cells to knock down its expression (Fig. 2A). As expected, si-circ_0018289 transfection inhibited circ_0018289 expression by > twofold (Fig. 2A). Analyses of CCK-8 and Edu showed that circ_0018289 loss of function remarkably impeded cell proliferation (Fig. 2B, C). Conversely, silencing endogenous circ_0018289 promoted cell apoptosis (Fig. 2D). Additionally, western blot analysis revealed that circ_0018289 knockdown increased pro-apoptotic protein Bax level and reduced anti-apoptotic protein Bcl-2 expression in both cell lines (Fig. 2E), reinforcing that circ_0018289 loss of function enhanced apoptosis.

### Circ_0018289 silencing suppressed angiogenesis in vitro

We next examined the influence of silencing endogenous circ_0018289 on angiogenesis in vitro. We firstly transfected SiHa and HeLa cells with si-circ_0018289 or nontarget-shRNA (si-NC) and then treated human HUVECs with the conditional medium of transfected cells. Strikingly, the incubation of conditional medium of cells with circ_0018289 loss of function led to an inhibition of tube formation of HUVECs (Fig. 2F), demonstrating that circ_0018289 silencing suppressed angiogenesis in vitro. Additionally, circ_0018289 knockdown decreased the levels of angiogenesis inducers VEGFA and FGF2 in the two cancer cell lines (Fig. 2G).

### Circ_0018289 directly targeted miR-183-5p

**Fig. 3:**
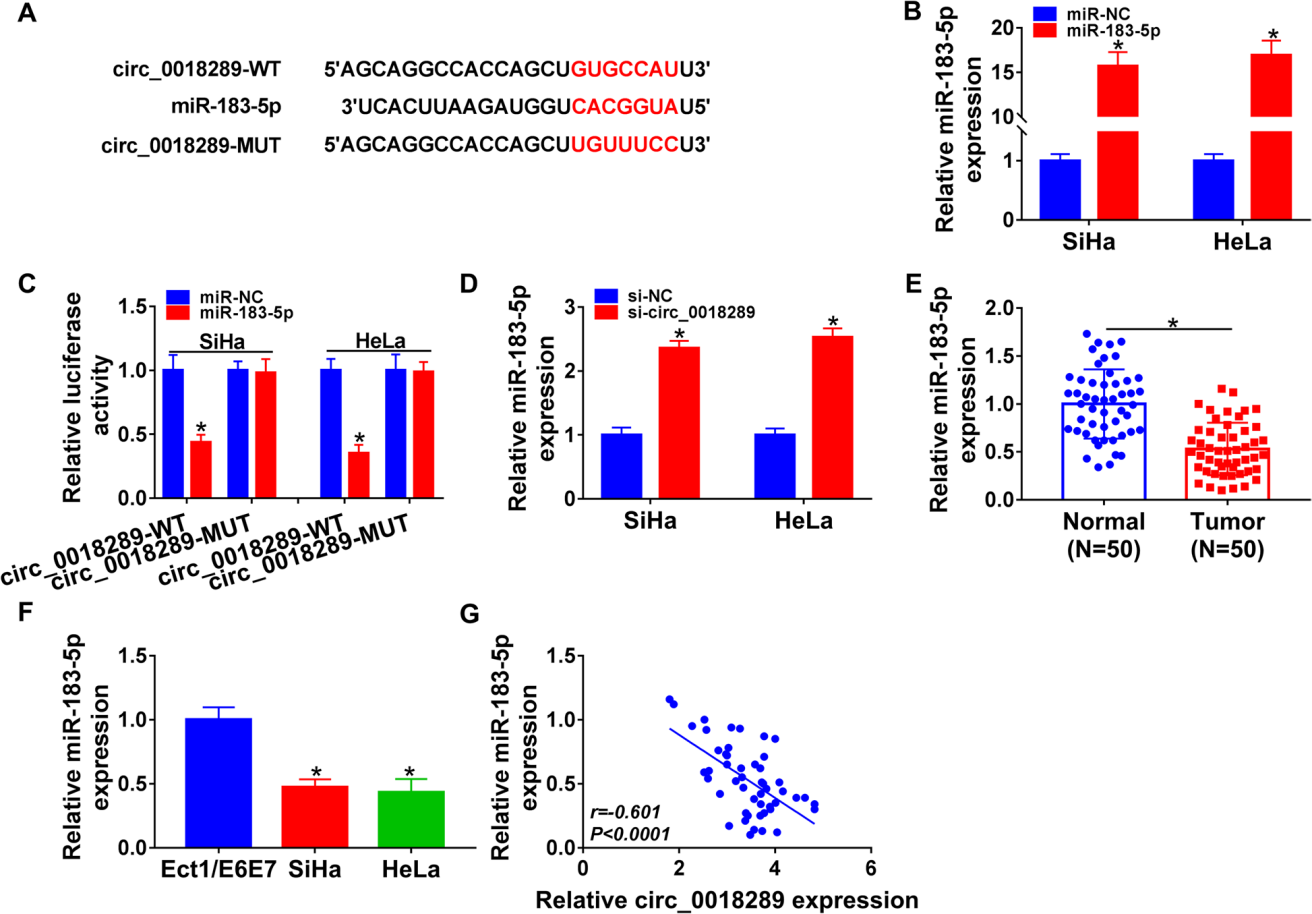
Circ_0018289 targeted miR-183-5p. **A** Sequence of miR-183-5p, the complementary sites for miR-183-5p in circ_0018289, and the mutant of the seed sites. **B** qRT-PCR analysis of miR-183-5p in miR-183-5p mimic- or miR-NC mimic-transfected SiHa and HeLa cells. **C** Dual-luciferase reporter assays using circ_0018289 wild-type (circ_0018289-WT) or mutant-type (circ_0018289-MUT) reporter constructs in SiHa and HeLa cells. **D** qRT-PCR analysis of miR-183-5p in si-circ_0018289- or si-NC-transfected SiHa and HeLa cells. **E** qRT-PCR analysis showing the expression of miR-183-5p in 50 primary tumor samples and 50 paired normal tissues from the same patients. **F** Relative miR-183-5p expression in Ect1/E6E7, SiHa and HeLa cells by qRT-PCR analysis. **G** Correlation between circ_0018289 and miR-183-5p levels in tumor samples analyzed by Pearson’s correlation coefficients. *P < 0.05

To further elucidate the mechanism by which circ_0018289 regulated cervical carcinogenesis and angiogenesis, we identified its targeted miRNAs. Computer algorithm circInteractome predicted that circ_0018289 harbored a region that was partially complementary to miR-183-5p (Fig. 3A). To verify this finding, we cloned circ_0018289 segment containing the seed sequence into a luciferase vector and cotransfected the reporter into SiHa and HeLa cells with miR-183-5p mimic. The transfection efficiency of miR-183-5p mimic was examined by qRT-PCR (Fig. 3B). Remarkably, the reporter construct with the binding sites for miR-183-5p was repressed by miR-183-5p overexpression (Fig. 3C). We then generated a mutant in the binding sequence (Fig. 3A) and tested it. As expected, the mutant encompassing a mutated binding region was refractory to repression of miR-183-5p (Fig. 3C). Moreover, we observed a clear augmentation (> twofold) in the level of the endogenous miR-183-5p in circ_0018289-silencing cells (Fig. 3D). Contrary to the level of circ_0018289, miR-183-5p was markedly underexpressed in tumor samples and cancer cells compared with their counterparts (Fig. 3E, F). Intriguingly, there existed an inverse correlation between miR-183-5p and circ_0018289 levels in tumor samples (Fig. 3G). These data together indicated that circ_0018289 targeted miR-183-5p by directly binding to miR-183-5p.

### MiR-183-5p mediated the regulation of circ_0018289 in cervical cancer development and angiogenesis in vitro

**Fig. 4:**
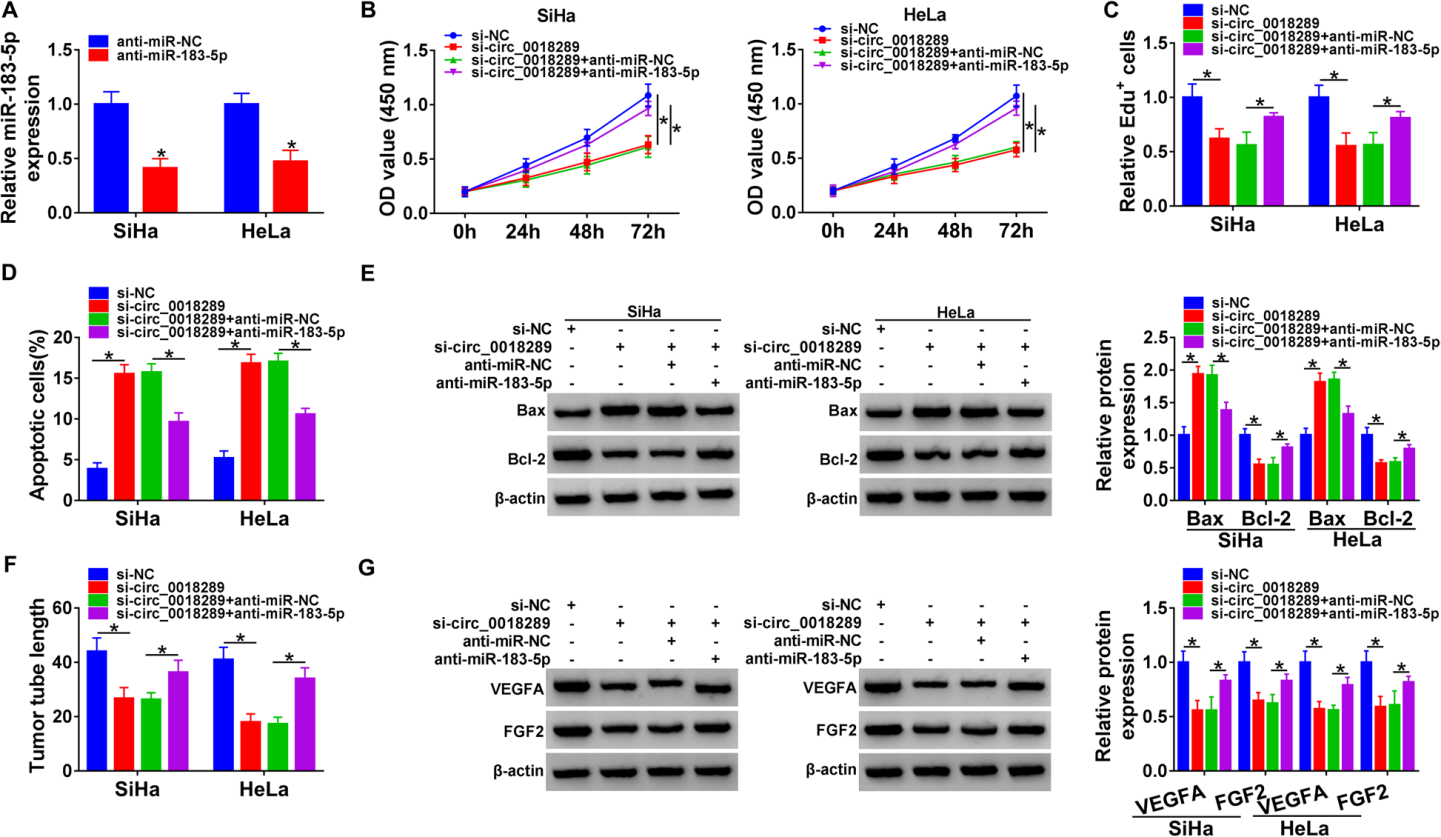
Silencing of circ_0018289 impeded cervical carcinogenesis and angiogenesis by up-regulating miR-183-5p. **A** qRT-PCR analysis of miR-183-5p in SiHa and HeLa cells transfected with anti-miR-183-5p or anti-miR-NC. CCK-8 (**B**) and Edu (**C**) assays for cell proliferation, flow cytometry for cell apoptosis (**D**), western blot analysis for Bax and Bcl-2 levels (**E**), in SiHa and HeLa cells transfected with si-circ_0018289, si-NC, si-circ_0018289 + anti-miR-NC or si-circ_0018289 + anti-miR-183-5p. **F** Tube formation assay performed with human HUVECs pre-treated with the conditional medium of SiHa and HeLa cells transfected with si-circ_0018289, si-NC, si-circ_0018289 + anti-miR-NC or si-circ_0018289 + anti-miR-183-5p. **G** Western blot showing the levels of VEGFA and FGF2 in SiHa and HeLa cells transfected with si-circ_0018289, si-NC, si-circ_0018289 + anti-miR-NC or si-circ_0018289 + anti-miR-183-5p. *P < 0.05

We next investigated whether circ_0018289 regulated cervical carcinogenesis and angiogenesis by miR-183-5p. The transfection efficiency of anti-miR-183-5p in SiHa and HeLa cells was confirmed by qRT-PCR (Fig. 4A). As expected, the transfection of anti-miR-183-5p significantly abrogated circ_0018289 silencing-mediated proliferation suppression (Fig. 4B, C) and apoptosis enhancement (Fig. 4D, E). Moreover, miR-183-5p depletion abolished the inhibition of circ_0018289 silencing on tube formation of HUVECs (Fig. 4F). Additionally, the deficiency of miR-183-5p counteracted the suppression of VEGFA and FGF2 expression of circ_0018289 knockdown in both SiHa and HeLa cell lines (Fig. 4G).

### TMED5 was directly targeted and inhibited by miR-183-5p

**Fig. 5:**
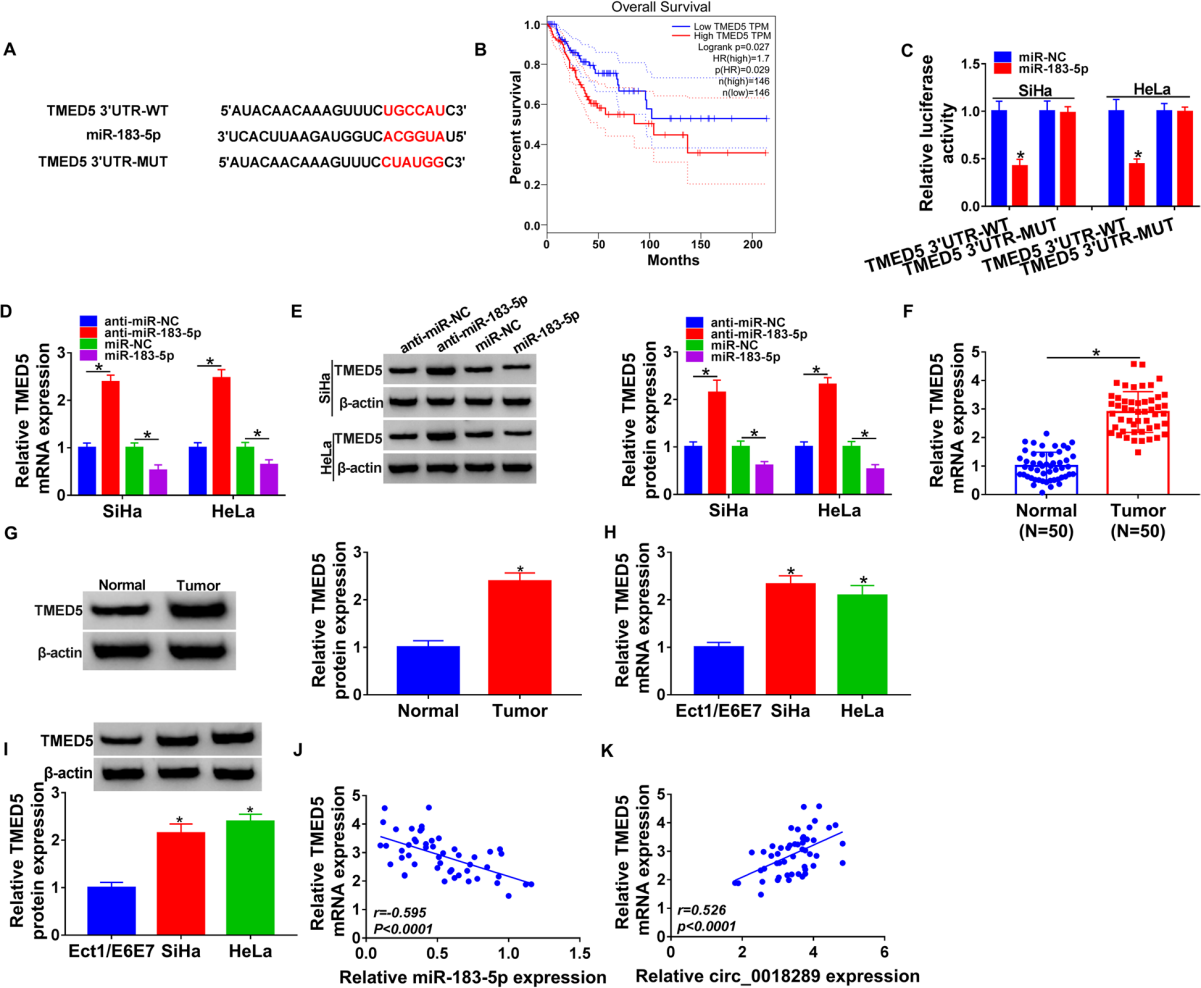
TMED5 was a direct target of miR-183-5p. **A** Sequence of miR-183-5p, the complementary sites for miR-183-5p in TMED5 3′UTR and the mutant of the seed sites. **B** Correlation between TMED5 level and the prognosis of patients with cervical cancer shown in the GEPIA database. **C** Dual-luciferase reporter assays using TMED5 3′UTR wild-type (TMED5 3′UTR-WT) and mutant (TMED5 3′UTR-MUT) reporter constructs in SiHa and HeLa cells. **D**, **E** TMED5 mRNA and protein levels in SiHa and HeLa cells transfected with anti-miR-NC, anti-miR-183-5p, miR-NC mimic, or miR-183-5p mimic by qRT-PCR and western blot assays, respectively. **F**, **G** TMED5 mRNA and protein levels in primary tumor samples and paired normal tissues from the same patients. **H**, **I** TMED5 mRNA and protein levels by qRT-PCR and western blot assays, respectively, in Ect1/E6E7, SiHa, and HeLa cells. **J**, **K** Correlation between TMED5 mRNA level and miR-183-5p expression or circ_0018289 expression in tumor samples analyzed by Pearson’s correlation coefficients. *P < 0.05

To identify downstream targets of miR-183-5p, we used computational prediction software starBase. Interestingly, a putative binding region for miR-183-5p was found in the 3’UTR of TMED5 (Fig. 5A), which was shown to be associated with the prognosis of cervical cancer patients using the GEPIA database (http://gepia.cancer-pku.cn/, Fig. 5B). To establish the direct relationship between TMED5 and miR-183-5p, we generated TMED5 3′UTR wild-type (TMED5 3′UTR-WT) and mutant (TMED5 3′UTR-MUT) reporter constructs and analyzed them by dual-luciferase reporter assays. Overexpression of miR-183-5p by miR-183-5p mimic transfection remarkably repressed reporter gene expression of TMED5 3′UTR-WT, and the mutation abrogated the suppression of miR-183-5p (Fig. 5C). Moreover, endogenous TMED5 mRNA and protein levels were strongly promoted by miR-183-5p depletion and inhibited as a result of miR-183-5p overexpression in the two cancer cell lines (Fig. 5D, E). Analysis of TMED5 expression in cervical cancer showed that TMED5 mRNA and protein levels were significantly promoted in tumor samples and cancer cells compared with the corresponding normal controls (Fig. 5F–I). Importantly, TMED5 mRNA level inversely correlated with miR-183-5p expression and positively correlated with circ_0018289 expression in tumor samples (Fig. 5J, K). All these data established the notion that miR-183-5p regulated TMED5 expression by the perfect binding sites in the 3′UTR.

### MiR-183-5p-mediated inhibition of TMED5 impacted cervical cancer development and angiogenesis in vitro

**Fig. 6:**
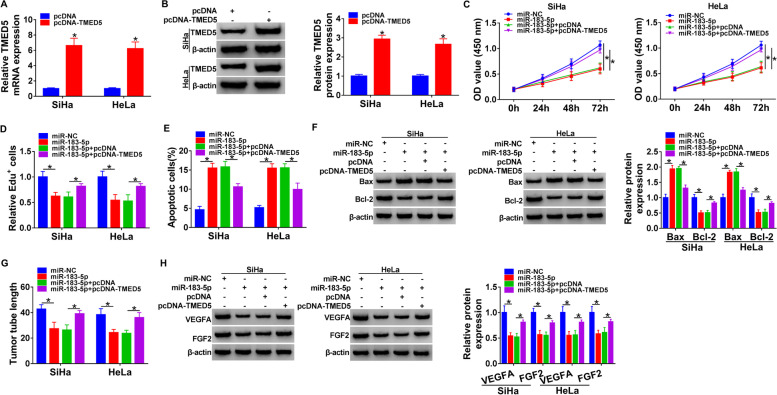
MiR-183-5p-mediated inhibition of TMED5 impeded cervical cancer development and angiogenesis in vitro. **A**, **B** TMED5 mRNA and protein levels by qRT-PCR and western blot assays, respectively, in SiHa and HeLa cells transfected with pcDNA-TMED5 or negative control pcDNA. SiHa and HeLa cells were transfected with miR-183-5p mimic, miR-NC mimic, miR-183-5p mimic + pcDNA, or miR-183-5p mimic + pcDNA-TMED5, followed by the assessment of cell proliferation by CCK-8 (**C**) and Edu (**D**) assays, cell apoptosis by flow cytometry (**E**), and Bax and Bcl-2 levels by western blot (**F**). **G** Tube formation assay performed with human HUVECs pre-treated with the conditional medium of SiHa and HeLa cells transfected with miR-183-5p mimic, miR-NC mimic, miR-183-5p mimic + pcDNA, or miR-183-5p mimic + pcDNA-TMED5. **H** Western blot showing VEGFA and FGF2 levels in SiHa and HeLa cells transfected with miR-183-5p mimic, miR-NC mimic, miR-183-5p mimic + pcDNA, or miR-183-5p mimic + pcDNA-TMED5. *P < 0.05

In order to demonstrate whether inhibition of TMED5 by miR-183-5p was responsible for the regulatory effects of miR-183-5p on cervical carcinogenesis and angiogenesis, we adopted a rescue experiment by increasing TMED5 expression in SiHa and HeLa cells. The transfection efficiency of TMED5 overexpression plasmid (pcDNA-TMED5) was gauged by qRT-PCR and western blot assays (Fig. 6A, B). As would be expected, elevated expression of TMED5 significantly abrogated miR-183-5p overexpression-mediated anti-proliferation (Fig. 6C, D) and pro-apoptosis (Fig. 6E, F) effects in the two cancer cell lines. Furthermore, elevated expression of TMED5 reversed the suppression of tube formation of miR-183-5p overexpression in HUVECs (Fig. 6G). Additionally, elevated expression of TMED5 abrogated miR-183-5p overexpression-mediated inhibition on VEGFA and FGF2 levels in both SiHa and HeLa cell line (Fig. 6H).

### Circ_0018289 regulated TMED5 expression by targeting miR-183-5p

**Fig. 7:**
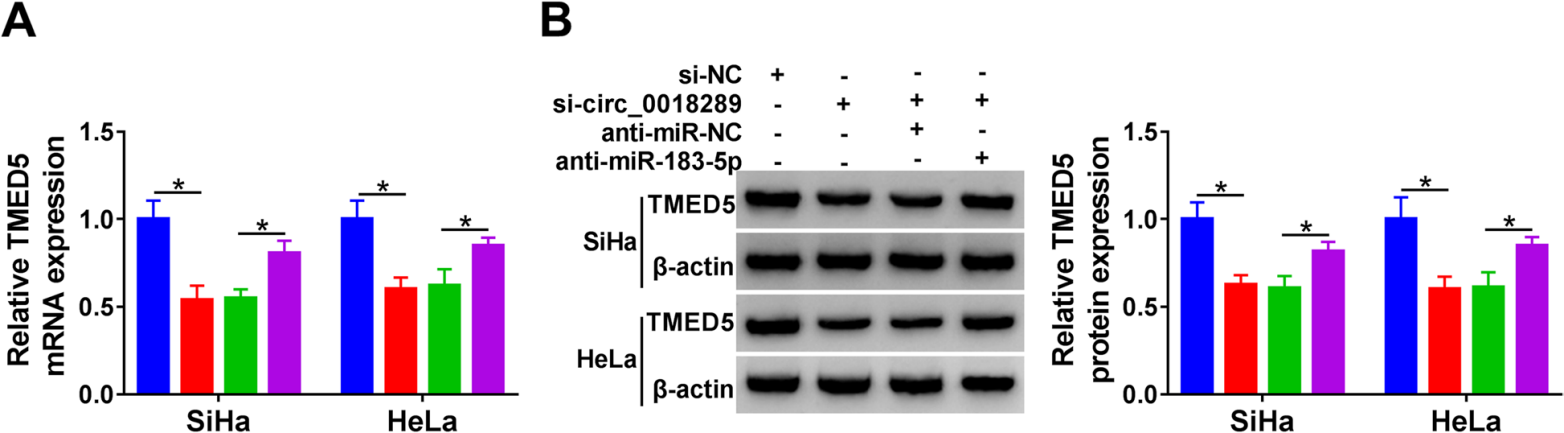
Circ_0018289 modulated TMED5 expression through binding to miR-183-5p. SiHa and HeLa cells were transfected with si-circ_0018289, si-NC, si-circ_0018289 + anti-miR-NC or si-circ_0018289 + anti-miR-183-5p. **A** qRT-PCR analysis of TMED5 mRNA level in transfected cells. **B** Western blot showing TMED5 protein level in transfected cells. *P < 0.05

Next, we asked whether circ_0018289 could operate as a regulator of TMED5. Notably, circ_0018289 loss of function resulted in reduced levels of TMED5 mRNA and protein in SiHa and HeLa cells (Fig. 7A, B). However, this effect was remarkably abolished by miR-183-5p depletion (Fig. 7A, B).

### Circ_0018289 silencing diminished tumor growth in vivo

**Fig. 8:**
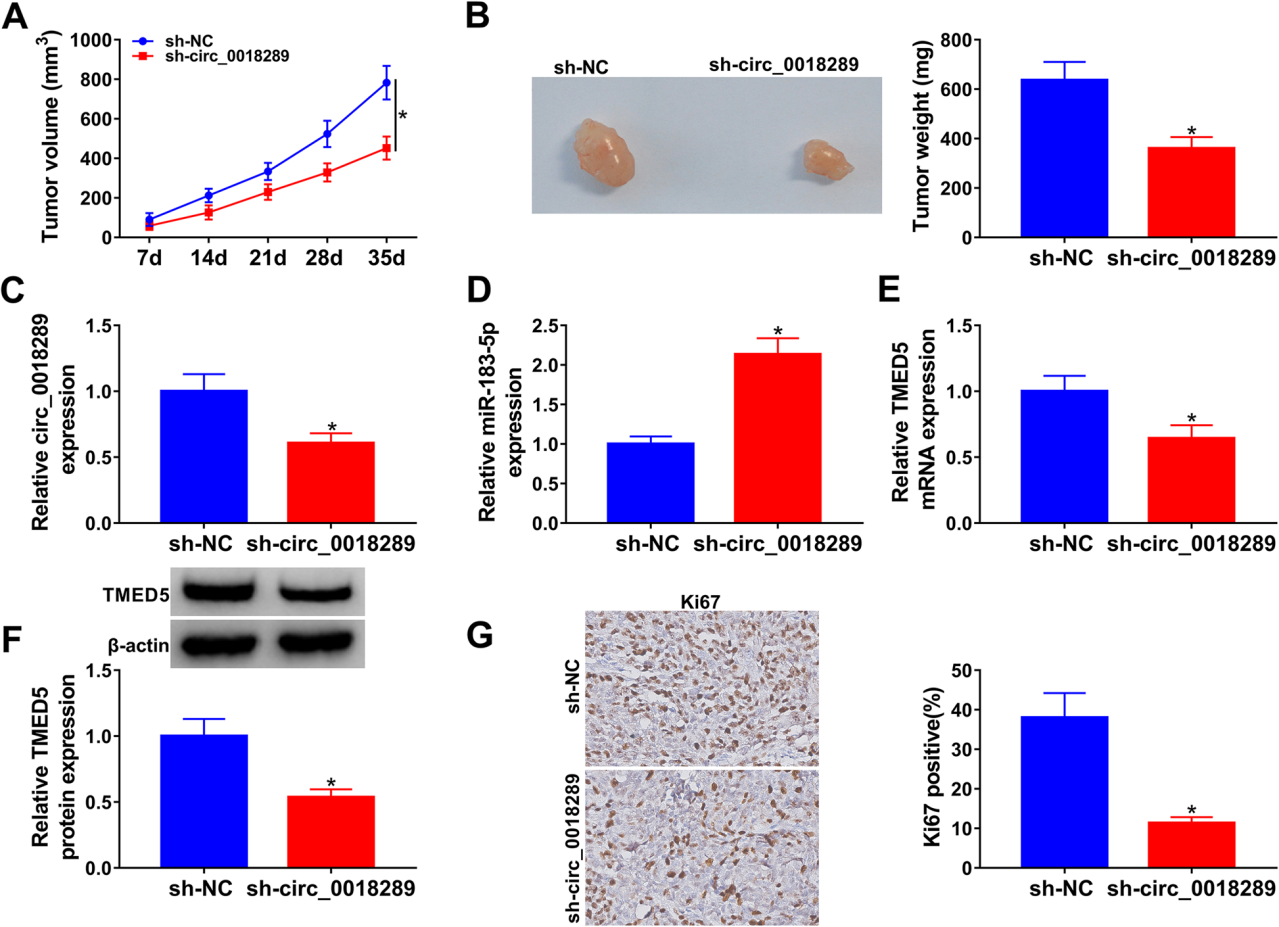
Circ_0018289 silencing weakened tumor growth in vivo. **A** Growth curves of the xenograft tumors derived from subcutaneous injections into the nude mice of sh-circ_0018289-transduced or sh-NC-infected SiHa cells (*n* = 6 per group). Representative images and average weight (**B**), circ_0018289 expression by qRT-PCR analysis (**C**), miR-183-5p expression by qRT-PCR analysis (**D**), TMED5 mRNA level by qRT-PCR analysis (**E**), TMED5 protein level by western blot (**F**), and Ki67 level by immunohistochemistry (**G**) of the xenograft tumors at day 35 formed by sh-circ_0018289-transduced or sh-NC-infected SiHa cells (*n* = 6 per group). *P < 0.05

To determine whether circ_0018289 silencing possessed tumor-inhibitory effects, we reduced circ_0018289 expression with a lentiviral delivery system (sh-circ_0018289) in SiHa cells and then implanted the cells subcutaneously in the nude mice. By contrast, transduction with sh-circ_0018289 led to markedly decreased volume and weight of the tumors (Fig. 8A, B). Moreover, the tumors formed by sh-circ_0018289-transduced SiHa cells exhibited lower levels of circ_0018289 compared with the controls (Fig. 8C). qRT-PCR and western blot analyses of the tumors showed that circ_0018289 silencing resulted in increased expression of miR-183-5p (Fig. 8D) and reduced levels of TMED5 mRNA and protein (Fig. 8E, F). Additionally, tumor-inhibitory result of circ_0018289 silencing was also confirmed by staining for cell proliferation using the cell-cycle marker Ki67 (Fig. 8G). These results together suggested that the inhibition of tumor growth might be due to downregulation of circ_0018289 and TMED5 and upregulation of miR-183-5p.

## Discussion

CircRNAs are increasingly implicated in regulating human carcinogenesis [16–18]. Previous work uncovered the overexpression and tumor-promoting activity of circ_0018289 in cervical cancer [10, 11]. We here extended these findings by identifying a novel circ_0018289/miR-183-5p/TMED5 regulatory network in cervical cancer development. Taken together, we proposed that circ_0018289 might be a potent oncogene in cervical tumorigenesis. As previously reported for other circRNAs [19, 20], circ_0018289 was resistant to RNase R due to the back-spliced formation and the lack of 3′ and 5′ ends [4]. Additionally, circ_0018289 was mainly present in the cytoplasm, providing the possibility for its relationship with mature miRNAs [21].

Emerging evidence has suggested that alterations in miRNA expression might prove crucial in affecting cervical tumorigenesis [22–24]. Here, we focused on miR-183-5p because of the conflicting roles of miR-183-5p in human carcinogenesis [25–27]. These contradictory conclusions might partially due to the different types of tumors in these reports, where miR-183-5p facilitated the development of breast cancer [26] and hepatocellular carcinoma [27] and impeded endometrial tumorigenesis [25]. Here, we first elucidated that circ_0018289 directly targeted miR-183-5p, which was established as a strong suppressor in cervical cancer [12, 13]. Our data also showed the regulation of circ_0018289 in cervical cancer development and angiogenesis through miR-183-5p.

TMED5, located at chromosomal region 1p21-22, was associated with the pathogenesis of myeloma and bladder cancer [28, 29]. A previous study uncovered the tumor-promoting effect of TMED5 on the malignant development of cervical cancer by activating the Wnt7b/β-catenin signaling pathway [30]. Our results identified that TMED5 was directly targeted and inhibited by miR-183-5p, and TMED5 knockdown phenocopied miR-183-5p overexpression in suppressing cervical cancer development and angiogenesis. Importantly, we highlighted that circ_0018289 induced TMED5 expression by binding to miR-183-5p. Furthermore, our data showed that the circ_0018289/miR-183-5p/TMED5 axis contributed to an elevated expression of VEGFA and FGF2, two angiogenesis inducers [31, 32], leading to induction of tumor-associated angiogenesis. Additionally, in vivo assays implied the involvement of the circ_0018289/miR-183-5p/TMED5 regulatory network in tumor growth, and the direct evidence should be further elucidated in further work.

## Conclusions

Taken together, our findings identified a novel molecular basis, circ_0018289/miR-183-5p/TMED5 regulatory network, underlying the modulation of cervical carcinogenesis. These observations suggested that circ_0018289 inhibition might be a promising point for the development of novel anti-tumor strategies against cervical cancer.

## Supplementary Information

**Additional file 1: Supplement Table 1.** Sequences of qRT-PCR primers and oligonucleotides.

## Data Availability

Not applicable.
