# Supplementary material for: Identification of a novel circ_0018289/miR-183-5p/TMED5 regulatory network in cervical cancer development
Source: World J Surg Oncol. 2021 Aug 17;19:246. doi: 10.1186/s12957-021-02350-y (PMC8371901; doi:10.1186/s12957-021-02350-y)
Supplement: Supplementary file 1 — Additional file 1: Supplement Table 1. Sequences of qRT-PCR primers and oligonucleotides. [file 12957_2021_2350_MOESM1_ESM.docx]

**Supplement Table 1. Sequences of qRT-PCR primers and oligonucleotides**

| Sequence (5’-3’) | | |
| --- | --- | --- |
| circ_0018289 | Forward | CCTGCACAGTGGAGAGTGG |
|  | Reverse | TCTCCACAGACAGCAGCTTG |
| SYT15 | Forward  Reverse | TCTGTGCCACCCTCACCTAT  GCTCAGTTGGGTCCTAGCAT |
| TMED5 | Forward  Reverse | GTGCAAATAAGACTGCATCTTGGA  TTGAATGGCTGACACCACCA |
| miR-183-5p | Forward | GCCGAGTATGGCACTGGTAG |
|  | Reverse | CAGTGCGTGTCGTGGAGT |
| GAPDH | Forward | GACAGTCAGCCGCATCTTCT |
|  | Reverse | GCGCCCAATACGACCAAATC |
| β-actin | Forward | CTCGCCTTTGCCGATCC |
|  | Reverse | GGGGTACTTCAGGGTGAGGA |
| U6 | Forward | CTCGCTTCGGCAGCACA |
|  | Reverse | AACGCTTCACGAATTTGCGT |
| si-circ_0018289 | UCGCCGCCGGAACUCGUCGAC | |
| si-NC | AAGACAUUGUGUGUCCGCCTT | |
| miR-183-5p mimic | UAUGGCACUGGUAGAAUUCACU | |
| miR-NC mimic | ACGUGACACGUUCGGAGAATT | |
| anti-miR-183-5p | AGUGAAUUCUACCAGUGCCAUA | |
| anti-miR-NC | CAGUACUUUUGUGUAGUACAA | |
